# The effects of color and saturation on the enjoyment of real-life images

**DOI:** 10.3758/s13423-023-02357-4

**Published:** 2023-08-24

**Authors:** Chenyang Lin, Sabrina Mottaghi, Ladan Shams

**Affiliations:** 1 Neuroscience Interdepartmental Program, University of California, Los Angeles, CA USA; 2 Department of Psychology, University of California, Los Angeles, CA USA; 3 Department of BioEngineering, University of California, Los Angeles, CA USA

**Keywords:** Color, Image perception, Affective judgment, Chromatic, Grayscale, Saturation, Valence, Aesthetic value, Perceptual pleasure, Affective value

## Abstract

This study investigated the effects of color presence and saturation on the affective judgment of real-life images, as functions of the image’s affective valence. In the first two experiments, participants observed and rated original color photos and their grayscale versions, presented in an interleaved order across two separate experimental sessions. Color photos were rated as more pleasant than grayscale photos when image valence was positive, and more unpleasant when image valence was negative. The third experiment consisted of the same original images and their versions with saturation reduced by 50%. Original photos were rated as more pleasant than saturation-reduced photos when image valence was positive, yet less unpleasant when image valence was negative, implying potentially separable mechanisms for processing color presence and saturation. Significant interactions were found between color or saturation mode and valence on affective judgment in all three experiments. The effects persisted after controlling for colorfulness and luminance between the color and grayscale (or de-saturated) conditions.

**Supplementary information:**

The online version contains supplementary material available at 10.3758/s13423-023-02357-4.

## Introduction

The human retina is populated with photoreceptors. The cones densely occupy the fovea, enabling color vision under photopic conditions. Human beings are able to perceive the world with an expansive palette. Normal chromats are capable of visualizing up to 2.3 million discernible colors (Linhares et al., 2008), which lead to an almost endless constellation of color combinations (Hard & Sivik, 2001). Numerous studies have demonstrated the direct association between color perception and emotion (Adams & Osgood, 1973; Boyatzis & Varghese, 1994; Crozier, 1999; Gilbert et al., 2016; Hemphill, 1996; Jacobs & Suess, 1975; Kaya & Epps, 2004; Valdez & Mehrabian, 1994; Wilms & Oberfeld, 2018). For instance, Kaya and Epps (2004) revealed that principal hues (e.g., red, yellow) evoked higher positive emotions than intermediate hues (e.g., yellow-red, blue-green) and achromatic colors (e.g., white, gray). The strong influence of saturation on the emotional processing of colors has also been repeatedly reported (Dael et al., 2016; Valdez & Mehrabian, 1994). Specifically, higher saturation has been reported to be associated with higher valence and arousal (Wilms & Oberfeld, 2018). However, Dael et al. (2016) suggest that saturation interacts with other color attributes, such as hue and brightness, in influencing affective processing. Conversely, a study by Lee and Andrade (2010) showed that affective states triggered by emotionally laden movies influence color preference; more specifically, positive moods increase the preference for long-wavelength colors, such as red and yellow. The influence of colors on human affect has implications in various spheres of decision making, including architectural design (Manav, 2017; Mahnke, 1996) and consumer marketing (Cunningham, 2017; Ettis, 2017).

Several theories have been proposed regarding color preferences. The evolutionary adaptive theory suggests that color preferences arise from adaptations in nature; the visual system for color processing has evolved to improve performance in evolutionarily important tasks (e.g., the redness of blood makes us alarmed), resulting in corresponding genetic changes that make us prefer one color over another (Humphrey, 2019; Hurlbert and Ling, 2007). According to the “color-emotion” theory, color preference is based on the emotions evoked by different colors (Ou et al., 2004a, 2004b, 2004c). Ou et al. found that the majority of color preference data could be explained by three dimensions of color-emotion – active/passive, heavy/light, warm/cool – with preferences found for active, light, and warm colors. Based on these theories, Palmer and Schloss (2010) raised the “ecological valence theory” (EVT), which proposed that color preferences are adaptive on an ontogenetic level. According to EVT, colors are perceived as either good or bad based on their association with objects that affect an observer's well-being. The theory also emphasizes the role of environmental inputs in shaping an individual's color preferences. However, these theories have only been tested with pure uniform fields of color in the absence of shapes and objects, and it is unclear whether they can be applied to more complex visual scenes.

Not many studies have directly probed the effects of colors in real-life photos on affective judgment. Real-life visual perception involves a complex combination of colors, other object cues (such as shapes), and emotional cues (Cano et al., 2009; Kaufman & Lohr, 2002; Oliva & Schyns, 2000; Suk & Irtel, 2010). Therefore, real-life images provide testbeds for investigating the effects of color modes as a global property in the context of the images’ emotional values, which largely stem from the recognition and appraisal of the presented objects or scenes (Lu et al., 2015). Growing evidence has suggested that, in addition to the shape property, surface properties such as color could affect object recognition in high-level vision (Tanaka et al., 2001). A few studies examined the effects of color presentation on the processing of emotional contents in static images (Bekhtereva & Müller, 2017; Cano et al., 2009; Codispoti et al., 2012), suggesting that color plays an important role in affective image processing by contributing to visual segmentation and information extraction (Gegenfurtner & Rieger, 2000; Hansen & Gegenfurtner, 2009). Along this argument, one could reasonably argue that, rather than simply having higher valence ratings than achromatic colors in pure color perception (Wilms & Oberfeld, 2018), chromatic colors in affective images augment the image’s affectivity, whether the affect is positive or negative.

However, in one of the seminal studies using International Affective Picture System (IAPS) images, Bradley et al. (2001) compared color with grayscale images and found no significant difference in affective reactions, as was demonstrated by self-reports and physiological measurements. No interactions between color mode and image valence were found either. The authors argued that color played little role in affective reactions, as semantic information, rather than perceptual features, was the major contributor to motivational activation. A subsequent functional neuroimaging study (Bradly et al., 2003) further supported these findings by showing the same activation patterns whether the images were presented in color or in grayscale. However, in both of these studies, relatively small pools of stimuli and between-subject designs were used, rendering them low in power. The large inter-subject variability in ratings may have masked the effects of the color mode. In the present study, we aimed to examine the effects of color presence (i.e., chromatic vs. grayscale) and saturation on the affective processing of real-life images as functions of the image valence, using a much larger sample of stimuli and a within-subject design.

## Experiment 1: Online chromatic and grayscale images

The goal of this experiment was to examine the influence of color presence in the enjoyment of real-life images for both positive- and negative-valence contents. We manipulated color presence by comparing original color images with their grayscale versions for two sets of images, one with positive valence and one with negative valence.

### Method

#### Participants

A total of 33 subjects (26 females, ranging from 18 to 22 years of age, mean age = 19.87 years) participated in this experiment. Sample size was determined by a power analysis using an estimate of variance and effect size from preliminary data, and a power of 80%. The required sample sizes for different effects were in the range of 6–33. Participants were undergraduate students enrolled in psychology courses at the University of California, Los Angeles. They were compensated with course credits for participation. All participants reported to have normal or corrected-to-normal vision and had no previous history of epilepsy or head trauma. All participants provided a written informed consent and agreed to participate. This study was approved by the Institutional Review Board at the University of California, Los Angeles. Subjects were notified of the presence of images that might cause discomfort at the beginning of the study and were informed that they could withdraw from the study at any time during the experiment without any penalty.

#### Stimuli

Four hundred images – 200 with positive and 200 with negative valence – were selected from IAPS. IAPS has been broadly used to investigate emotion and attention, with standardized valence, arousal, and dominance ratings for each image (Lang et al., 2008) in scales of 1–9. Images with mean valence ratings of at least 5.5 were categorized as positive and those with mean valence ratings lower than 4.5 were categorized as negative. We selected 200 images with positive valence and 200 with negative valence. Images with ratings between 4.5 and 5.5 were not selected. The positive images selected had a mean rating of 6.90 (SEM = 0.046), and the negative images selected had a mean rating of 3.23 (SEM = 0.054). The image contents varied widely from romantic couples and natural sceneries to dangerous animals and fights with blood. The grayscale versions of all images were created, leading to a pool of 800 images (400 chromatic, 400 grayscale) in total.

#### Procedure

**Fig. 1:**
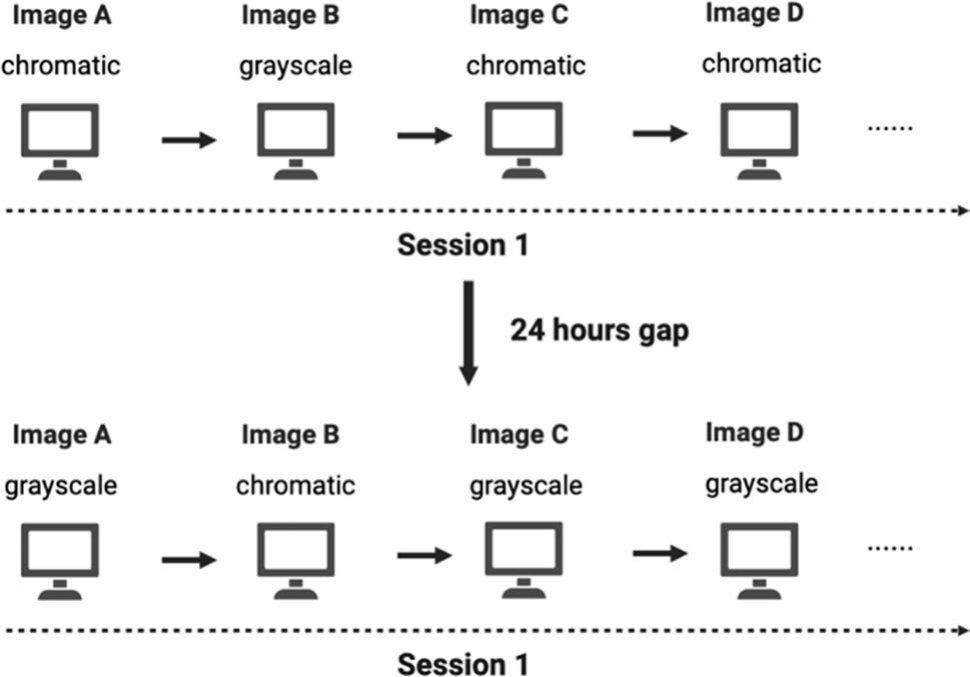
Design of Experiment 1. Each subject participated in two sessions, with a 24-h gap in between. The color mode for each image in session 1 was fully randomized and was reversed in session 2. The order of images within the session was the same across the two sessions

Each subject participated in two separate experimental sessions, separated by an approximately 24-h gap. To increase experimental power and reduce repetition effect, both versions of an image were presented to each participant but were presented separately in two sessions. In session one, participants were presented with all 400 different images in a random order. The color mode of each image was selected with a fully random process (rather than alternating between chromatic and grayscale across images). In session 2, the order of presented images was the same as the one in session 1. The only difference between the two sessions was that the color mode of each image was flipped so that an image was presented in the grayscale mode in session 2 if it was previously presented in the chromatic mode, and vice versa (see Fig. 1).

All images were presented on a black background for 1 s. After each presentation, subjects were asked to report how pleasant they found the image on a continuous scale. The scale was marked at the left end, the center, and the right end, respectively, as “Very Unpleasant”, “Neutral”, and “Very Pleasant,” corresponding to values of -100, 0, and 100. At the end of the second session, subjects were asked to complete a survey with demographic questions and questions regarding previous picture-editing experiences. Due to the ongoing COVID-19 pandemic, all experimental sessions were conducted online via Qualtrics (Qualtrics, Provo, UT, USA), an online survey platform, while the subjects were being monitored by an experimenter via Zoom (Version 5.4.7) with their cameras and speakers on and screens shared. The subjects were asked to use the same device and browser across the two experimental sessions and to keep the screen brightness identical at a comfortable level across sessions.

### Results

**Fig. 2:**
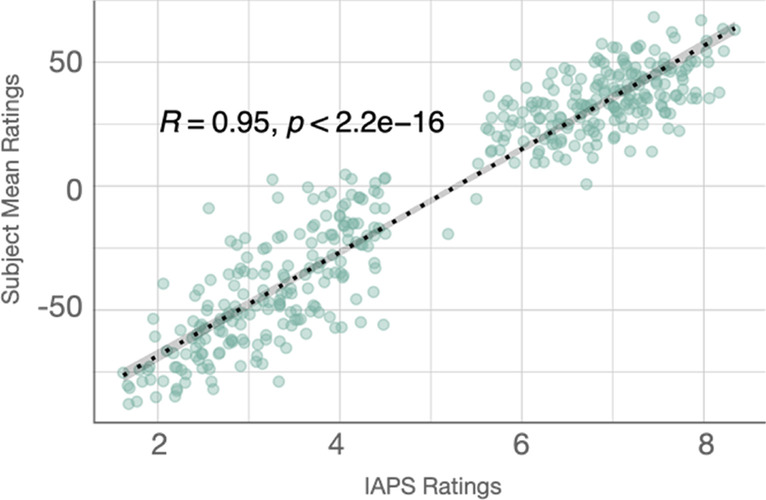
Correlation between the International Affective Picture System (IAPS) valence scores and the mean ratings for original images in Experiment 1. The IAPS valence scores and the mean ratings were strongly correlated, *r* = 0.946, *p* < 0.001

To examine the validity of our valence conditions, we calculated the correlation between the IAPS valence ratings for the selected images and the participants’ ratings for original images. A Pearson correlation coefficient of 0.946 (*p* < 0.001) supports the validity of our continuous rating scales (see Fig. 2).

A two-way repeated-measures ANOVA was performed to analyze the effects of valence and color mode on pleasure ratings, and significant interactions were found between valence and color mode (*F*(1, 32) = 51.41, *p* < 0.001). Additionally, results showed significant main effects of valence (*F*(1, 32) = 245.81, *p* < 0.001) and color mode (*F*(1, 32) = 24.33, *p* < 0.001).

**Fig. 3:**
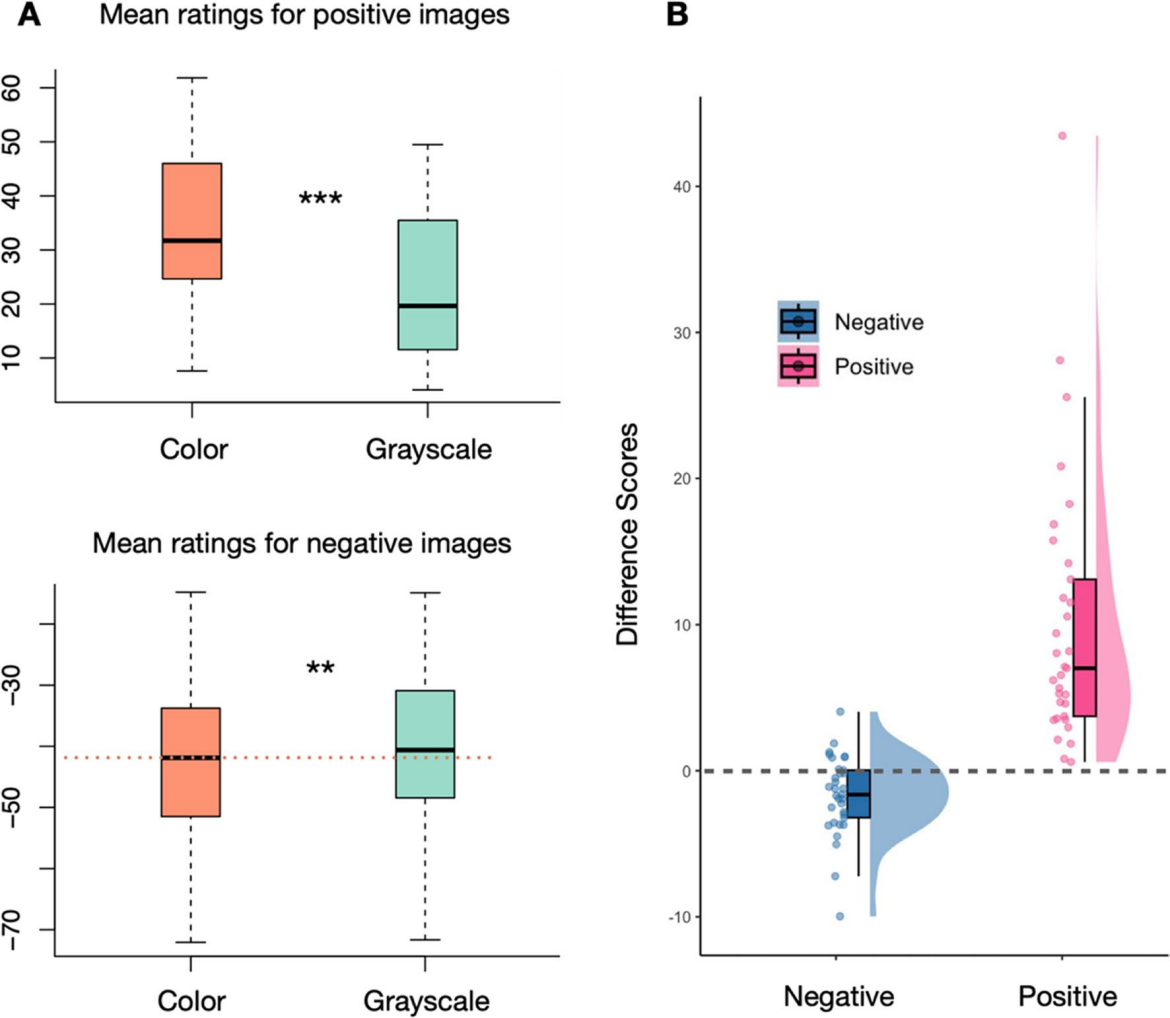
Experiment 1 results. (**A**) Mean pleasure ratings of two color conditions (color vs. grayscale) for positive images (top) and negative images (bottom). Significant differences between color modes were found in both positive and negative images. (**B**) Distribution of difference scores between color modes (color – grayscale) for negative and positive valence images

Paired two-tailed t-tests were conducted on subject-level mean ratings for both positive and negative images, with a Bonferroni-adjusted alpha level of 0.025 (.05/2) for each test, to confirm the effects. Cohen’s d was calculated using mean subject-level difference scores and their standard deviation (${Cohen}^{\prime }s\ {d}_z=\frac{\mu\ \left( difference\ score\right)}{SD\ \left( difference\ score\right)}$), according to the within-subject design of the experiment. For positive images, chromatic versions (*M* = 33.26, *SEM* = 2.58) had significantly higher ratings than grayscale versions (*M* = 23.24, *SEM* = 2.43) (*t*(32) = 6.30, *p* < 0.001, Cohen’s d_z_=1.10). For negative images, chromatic versions (*M* = -42.58, *SEM* = 2.94) had significantly lower ratings than grayscale versions (*M* = -40.87, *SEM* = 2.98) *(t* (32) = -3.60, *p* = 0.001, Cohen’s *d*_z_ = -0.63) (see Fig. 3).

Another correlation analysis was conducted between the IAPS valence ratings and the mean difference scores (color – grayscale), showing a moderate correlation of 0.650 (*p* < 0.001), further supporting the effects of valence properties of the images on pleasure ratings.

We next investigated whether the observed effects could have been due to any possible confounding factors, such as colorfulness or luminance differences across valence conditions. To this end, we computed the colorfulness and luminance of each image based on the metrics developed by Hasler and Suesstrunk (2003) and Morgand and Tamaazousti (2014), respectively (see Online Supplementary Material (OSM) for more details). Next, to equate the average colorfulness and luminance across valence conditions, we excluded 32 images from each of the two pools. The remaining 168 positive images and 168 negative images had equal mean colorfulness and luminance (see OSM). We then applied the same analyses as described above to the ratings of these smaller image pools and found qualitatively the same result (see Appendix B.1).

### Discussion

Experiment 1 investigated the effects of presence of color (chromatic vs. grayscale) on affective judgment of images with different valence attributes. Interactions were observed between valence and color presence on pleasure ratings. Compared to the grayscale condition, the chromatic condition amplified observers’ affective responses to both positive and negative images. This is in contrast to the study by Codispoti et al. (2012), in which a significant effect was only found for unpleasant pictures. All effects persisted in the repeated analysis after excluding images to equate the mean colorfulness and luminance between positive and negative images. We argue that this could be due to a lack of experimental power from the between-subject design and a relatively small number of stimuli in the study. Here we included a larger pool of images with an extensive variety of contents and utilized a within-subject design, which led to more experimental power.

Previous studies proposed that color presence contributes to the observer's extraction of the scene gist and facilitates the activation of conceptual representations (Castelhano & Henderson, 2008; , Goffaux et al., 2005; Oliva & Schyns, 2000). An electroencephalography study also found that color serves as a critical factor in increasing valence effects as demonstrated in the measured event-related potentials (Cano et al., 2009). Based on these findings, we argue that color presence facilitates the segmentation of the images and extraction of concepts to enhance emotional processing. Another interpretation of the current results would be that, instead of facilitating scene segmentation, color contributes to certain qualities of the presented objects (e.g., green vegetables are fresh and green snakes are toxic) that would enhance their emotional values.

## Experiment 2: In-Lab chromatic and grayscale images

Experiment 2 was a replication of Experiment 1 under better-controlled conditions. Experiment 1 was performed online. Therefore, the stimulus presentation devices such as computer monitor, CPU (central processing unit), graphic card, video card, as well as other environmental factors such as the levels of ambient light and the background noises varied across participants. At the time of Experiment 2, we were allowed to collect data on campus, and all sessions were conducted in the lab.

### Method

#### Stimuli and apparatus

Stimuli were presented using a Dell Desktop computer running Windows 7 (optiplex 7010) and a Dell flat screen LCD monitor. The resolution (1,920 x 1,080) and brightness (50%) of the screen were kept constant throughout the experiment. The room lighting was dim, and participants were seated approximately 35–40 cm from the screen. The images were presented at the center of the screen with a black background.

#### Participants

A total of 33 subjects (23 females, one non-binary, ranging from 17 to 23 years of age, mean age = 19.48 years) were recruited in this experiment. The sample size was determined by a power analysis using an estimate of variance and effect size from preliminary data, and a power of 80%. All other aspects of subject recruitment, consent, and inclusion criteria remained identical to Experiment 1.

#### Procedure

Each subject participated in two experimental sessions in the lab, separated by an approximately 24-h gap. Subjects were required to have masks on throughout the experiment. All sessions were conducted via Qualtrics (Qualtrics), an online survey platform, on the same device with controlled screen resolution and brightness. Subjects were monitored by an experimenter who sat right outside the experiment room. The rest of the procedure for stimuli presentations and pleasure ratings remained identical to that of Experiment 1.

### Results

A two-way repeated-measures ANOVA was performed to analyze the effects of valence and color mode on the pleasure ratings, and a significant interaction was found between valence and color mode (*F*(1, 32) = 62.20, *p* < 0.001). Additionally, results showed significant main effects of valence (*F*(1, 32) = 242.49, *p* < 0.001) and color mode (*F*(1, 32) = 25.35, *p* < 0.001).

**Fig. 4:**
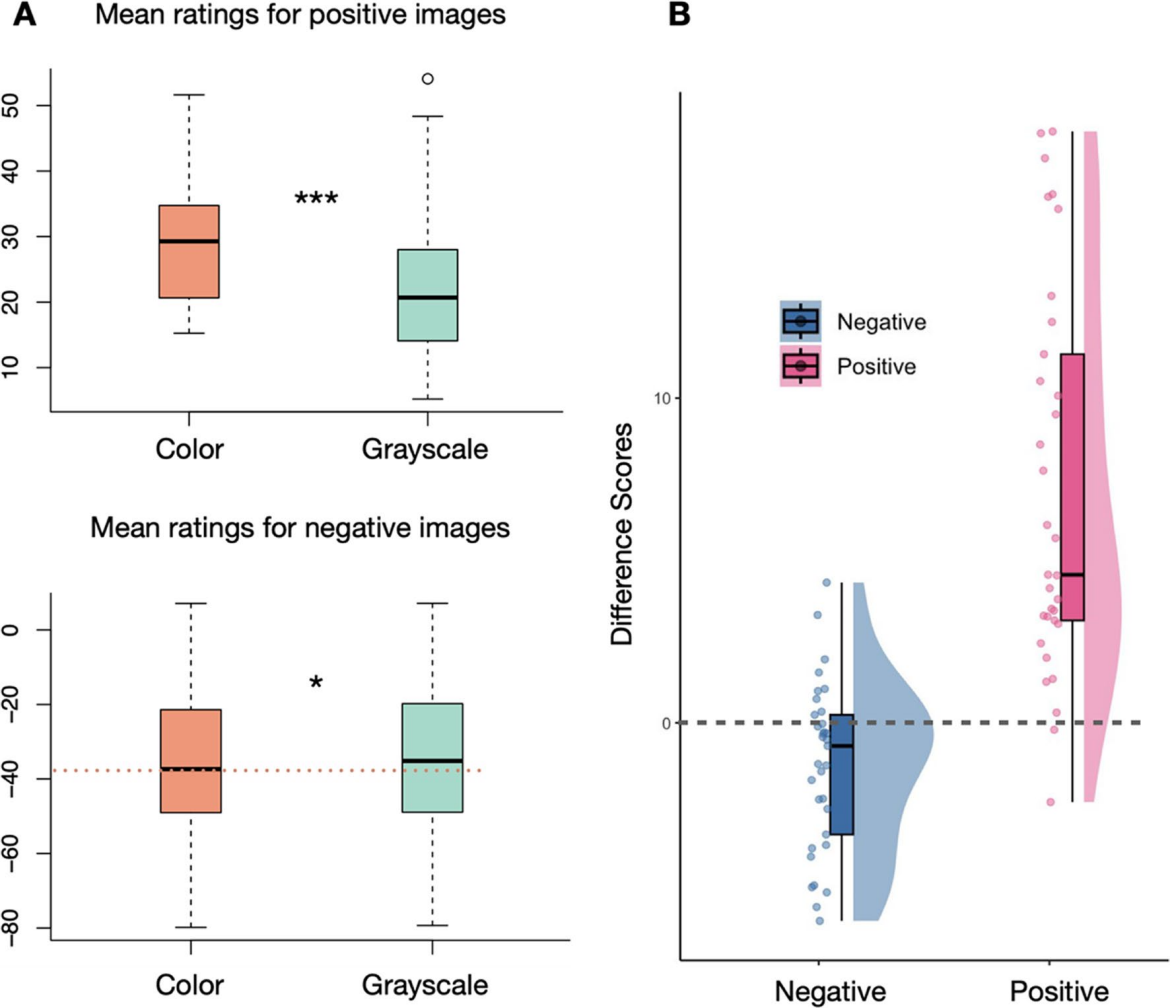
Experiment 2 results. (**A**) Mean pleasure ratings of two color conditions for positive images (top) and negative images (bottom). Significant differences between color modes were found in both positive and negative images. (**B**) Distribution of difference scores between color modes (color – grayscale) for negative and positive images

Paired two-tailed t-tests were conducted on subject-level mean ratings for both positive and negative images, with a Bonferroni-adjusted alpha level of 0.025 (.05/2) for each test, to confirm the effects. For positive images, chromatic versions (*M* = 29.50, *SEM* = 1.91) had significantly higher ratings than grayscale versions (*M* = 22.27, *SEM* = 1.89) (*t*(32) = 6.98, *p* < 0.001, Cohen’s *d*_*z*_ = 1.22). For negative images, chromatic versions (*M* = -35.35, *SEM* = 3.29) had significantly lower ratings than grayscale versions (*M* = -34.02, *SEM* = 3.25) (*t*(32) = -2.94, *p* = 0.006, Cohen’s *d*_*z*_ = -0.51) (see Fig. 4).

In addition, a Pearson correlation coefficient was computed in the post hoc analysis to assess the linear relationship between the IAPS ratings and the mean difference scores (color – grayscale) for the selected images in Experiment 2. The results showed a moderate correlation of 0.531 (*p* < 0.001), consistent with Experiment 1.

We next applied the same analyses to the reduced sets of images that were equated for luminance and colorfulness as in Experiment 1 and found qualitatively the same results (see Appendix B.2).

### Discussion

Experiment 2 provided a strong replication of Experiment 1. This replication supports the validity of the online experimental design in Experiment 1, suggesting that the online paradigm had comparable data quality in the context of the present study. Notably, for both positive and negative images, the effect sizes of color modes (color vs. grayscale) were consistent across Experiments 1 and 2, supporting the reliability of the online protocols.

### Experiment 3: Online intact and saturation-reduced images

The previous two experiments explored the effects of color presence (chromatic vs. grayscale) on the pleasure ratings of images. One approach to interpret the grayscale modes is that the saturation is perceptually set to 0%. However, we wanted to understand if human affective processing of real-life visual stimuli is associated with image saturation in a linear manner. In other words, are the affective responses to reduced-saturation images different between those to grayscale and to original images? In Experiment 3, we investigated this question using the same 400 original images and their 50% saturation-reduced versions. Based on previous findings on color enhancing affectivity, we expected a reduced affective response in the saturation-reduced condition (lower pleasure ratings for positive images and higher pleasure ratings for negative images).

### Method

#### Participants

A total of 33 subjects (24 females, ranging from 18 to 28 years of age, mean age = 20.09 years) were recruited in this experiment. The sample size was determined by a power analysis using an estimate of variance and effect size from preliminary data, and a power of 80%. All other aspects of subject recruitment, consent, and inclusion criteria remained identical to Experiment 1.

#### Stimuli

The 400 original chromatic images from Experiments 1 and 2 and their 50% saturation-reduced versions were used, leading to a pool of 800 images (400 original, 400 saturation-reduced) in total.

#### Procedure

Each subject participated in two separate experimental sessions, separated by an approximately 24-h gap. In session one, participants were presented with all 400 different images in a random order. The saturation of each image (original or reduced) was selected with a random process. In session 2, the same order of images was presented, with a flipped version of each image. At the end of session 2, besides demographic information and past experience with image editing, participants were also asked whether they had noticed any systematic patterns in the way the images were presented. The rest of the procedure for stimuli presentations and pleasure ratings remained identical to that of Experiment 1. The subjects were asked to use the same device and browser across the two experimental sessions and keep the screen brightness identical at a comfortable level across sessions.

### Results

A two-way repeated-measures ANOVA showed a significant interaction between valence and saturation (*F*(1, 32) = 9.64, *p* = 0.004). Additionally, results showed significant main effects of valence (*F*(1, 32) = 197.82, *p* < 0.001) and saturation (*F*(1, 32) = 26.53, *p* < 0.001).

**Fig. 5:**
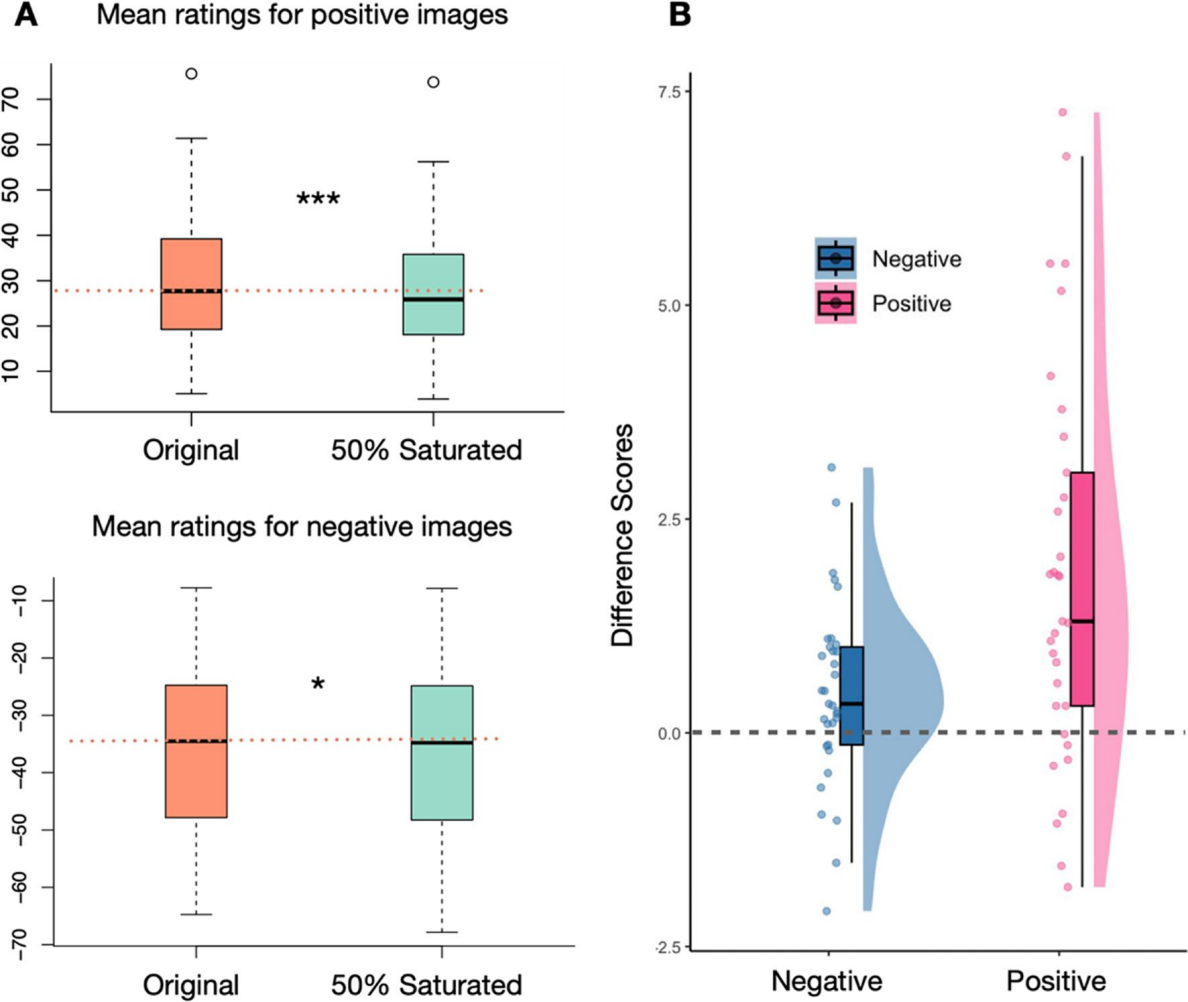
Experiment 3 results. (**A**) Mean pleasure ratings of two saturation conditions for positive images (top) and negative images (bottom). Significant differences between saturation conditions were found in both positive and negative images. (**B**) Distribution of mean difference scores (original – reduced) between saturation modes for negative and positive images

Paired two-tailed t-tests were conducted on subject-level mean ratings for both positive and negative images, with a Bonferroni-adjusted alpha level of 0.025 (.05/2) for each test, to confirm the effects. For positive images, original chromatic versions (*M* = 30.82, *SEM* = 2.76) had significantly higher ratings than saturation-reduced versions (*M* = 28.97, *SEM* = 2.63) (*t*(32) = 4.56, *p* < 0.001, Cohen’s *d*_*z*_ = 0.79). Surprisingly, for negative images, original chromatic versions (*M* = -36.53, *SEM* = 2.59) had significantly higher ratings than saturation-reduced versions (*M* = -36.99, *SEM* = 2.65) (*t*(32) = 2.43, *p* = 0.021, Cohen’s *d*_*z*_ = 0.42) (see Fig. 5).

Only four out of the 33 participants reported having observed a systematic difference in the way the pictures were presented (for instance, the brightness and contrast of the image or how prominent the color is in an image), and none had noticed the differences in saturation between conditions. This suggests that the manipulation of saturation remained largely subliminal to the subjects.

A Pearson correlation analysis showed a weak but significant correlation of 0.186 (*p* < 0.001) between the IAPS ratings and the mean difference scores (color – grayscale).

We next applied the same analyses to the reduced sets of images that were equated for luminance and colorfulness as in Experiment 1 and found qualitatively the same results (see Appendix B.3).

## General discussion

The three experiments explored the effects of color and saturation on the affective processing of real-life images, and whether it is influenced by the valence attributes of the images. Experiment 2 demonstrated a strong replication of Experiment 1, indicating that color presence amplifies the emotional reactions to real-life images, for both pleasant and unpleasant images. The effect of color on pleasure ratings was sizable, especially for positive images (Cohen’s d_z_ = 1.10 in Experiment 1; Cohen’s d_z_ = 1.22 in Experiment 2). This amplification effect could be due to the facilitation of image semantic processing by color. In other words, a more efficient processing, and thus a more unambiguous understanding, of pleasant content can lead to a stronger experience of pleasure, and a more unambiguous understanding of unpleasant content can result in a more aversive experience. Both the nature of the effect as well as the effect size were remarkably consistent between the online and in-lab experiment, supporting the reliability of our online experimental protocols.

In Experiment 3, we reduced the saturation of color images by 50%. This made the colors appear more pale while still being consciously perceived. Consistent with the first two experiments, the reduction of saturation led to the reduction of pleasure rating for positive images. However, in contrast to the first two experiments, pleasure ratings in negative images showed an advantage for the original chromatic condition. The fact that most participants in Experiment 3 did not report noticing the change in the image saturation, even having been explicitly asked at the end of Experiment 2, suggests that the influence of color saturation on affective judgment of real-life images may be subliminal.

**Fig. 6:**
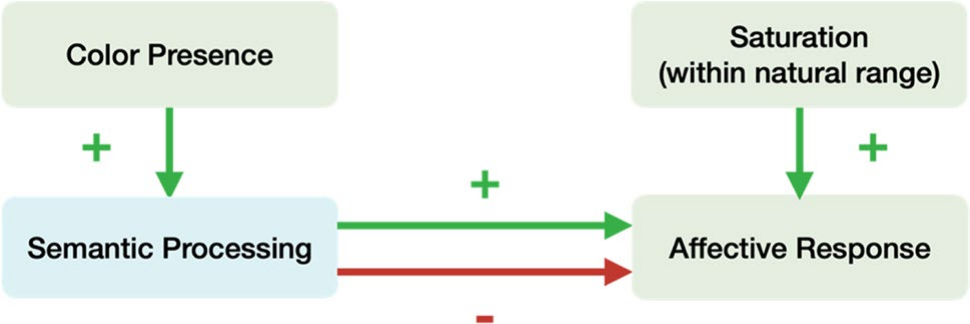
Schematic representation of the separable effects of color presence and saturation on affective response. Color presence enhances semantic processing, and thus the affectivity, of the visual stimuli, increasing the affective response to positive stimuli and decreasing the affective response to negative stimuli; however, saturation only increases affective response

The difference in the nature of interaction between valence and color in the experiments that manipulated the presence of color versus the experiment that manipulated the saturation may be due to separate affective processing mechanisms of color presence and saturation in human visual perception. While previous research argued that color presence could facilitate segmentation of and information extraction from visual stimuli, it remains unknown what level of saturation is enough for this effect (Gegenfurtner & Rieger, 2000; Hansen & Gegenfurtner, 2009; Saarela & Landy, 2012). We propose that, while the existence of color could facilitate scene segmentation and thus enhance the affective processing of images, saturation adds on another layer in that, consistent with previous findings of saturation’s effects on the affective response to uniform colors, reasonably higher saturation is associated with higher aesthetic pleasure (Wilms & Oberfeld, 2018). In other words, the presence of color augments the affectivity of images, whether it is positive or negative. However, once color is present, higher saturation, assuming it is within a reasonable range so as not to distort the images and make them unnatural, always leads to more positive (or less negative) affective responses (see Fig. 6).

Similarly, the same conceptual model for color presence and saturation could be applied to color presence and semantics-color congruence. Hekkert’s Principle of “maximum effects for minimum means” proposes that sensory stimuli that require a minimal amount of brain capacity for processing and that are perceptually fluent are preferred (Hekkert 2006). Many studies have also supported the connections between perceptual fluency and positive affect (Reber et al., 1998, 2004). When the saturation of an image is reduced, its semantic information may become incongruent with its color presentation, decreasing its perceptual fluency and aesthetic value. While color presence enhances image affectivity, whether positive or negative, decreased perceptual fluency caused by reduced saturation always leads to more negative (or less positive) affective responses.

This could be used to explain the results in all three experiments. In Experiments 1 and 2, while both color presence and saturation/semantics-color congruence enhance affective responses to positive images, the effect of color presence trumps that of saturation/semantics-color congruence in negative images, leading to a significantly lower rating (more unpleasant) for the chromatic condition. In Experiment 3, the effects of color presence and saturation/semantics-color congruence counteract and the effect of saturation/semantics-color congruence outweighs in the context of negative images. As a result, more positive (less unpleasant) ratings of the original chromatic images for negative images were observed.

Another way to interpret the results would be that different optimal saturation levels exist for positive and negative images. Yendrikhovski et al. (1998) proposed the model that the quality index in perception of real-life images is a linear combination of the perceived naturalness and colorfulness. Therefore, while generally an enhanced colorfulness (chroma) is preferred, how natural the image looks, affected by the saturation, could also contribute to the general quality, or aesthetic judgment, of the image. Along this argument, we propose that there are different optimal saturation levels for positive and negative images that could elicit their strongest corresponding affective responses, either positive or negative. For positive images, the optimal saturation level might be higher (e.g., original photos or more saturated). Therefore, more pleasantness was attributed to the chromatic or more saturated positive images. For negative images, the optimal saturation level might be lower (e.g., between 50% and 100%, but closer to 50%). Therefore, while original chromatic negative images were more unpleasant than grayscale ones, less saturated negative images were even more unpleasant.

All three experiments showed that color presence and saturation contribute to emotional reactions to real-life images. This finding is inconsistent with those of several previous studies, which argued that emotional reactivity is specific to the semantic content of the images and not influenced by perceptual features such as brightness and color (Bradley et al., 2001; Junghöfer et al., 2001). The current findings are more in line with the surface-plus-edge-based account, which postulates that object color facilitates object recognition, depending on the object's color diagnosticity or structural properties (Humphrey et al., 1994; Price & Humphreys, 1989; Tanaka et al., 2001; Tanaka & Presnell, 1999; Wurm et al., 1993). Additionally, our study found that reducing saturation amplified negative affective response. The findings altogether suggest that more than one mechanism may be involved in the quantitative effects of color saturation on affective responses, and warrant further investigation.

The current findings about the role of color in amplifying emotional valence of real-life scenes can inform appropriate usage of color in a variety of contexts. If the goal is to reduce the negative emotional toll on observers who have to view disturbing images or videos, the presenter may remove color and convert the images/videos to grayscale. For instance, in exposure-based therapies for fear or anxiety, grayscale visual stimuli may be utilized prior to introducing chromatic stimuli to facilitate a smoother transition for patients (Minns et al., 2019). In contrast, in educational or entertainment settings involving positive valence, color may be utilized to enhance the enjoyment of the content and better engage the viewer. For instance, the practice of color enhancement has been observed in classrooms to improve acquisition of textual information (Hall & Sidio-Hall, 1994) and anatomical learning experience (Inoue et al., 2020).

Future studies can supplement the subjective reports with physiological measurements to provide a more complete picture of affective processing of the stimuli in different conditions, and perhaps help delineate the role of image understanding and affective processing (Kuzinas et al., 2016). The role of saturation in pictorial affective responses could also be further explored by presenting observers with images of a spectrum of saturations. Additionally, in the current study participants were not asked whether they had any color deficiencies. Color deficiencies would reduce the magnitude of the differences observed between color and grayscale/saturation-reduced conditions. Therefore, to ascertain the effect sizes, future studies should inquire whether participants are color deficient.

## Conclusion

This study showed a sizable effect of color on amplifying the experience of pleasure and displeasure in real-life images. Color increased the pleasure ratings of pleasant images by as much as one standard deviation, and in unpleasant images it reduced the ratings by more than half a standard deviation. Additionally, we found a nonlinear relationship between saturation and pleasure ratings of the images, suggesting separable mechanisms for the effects of color presence and saturation on the affective response to real-life images.

### Supplementary Information

ESM 1 (PDF 2602 kb)

## Data Availability

The data for all experiments are available at https://osf.io/gu25n/.

## Appendix A

We quantified the perceived colorfulness of each image based on the metric developed by Hasler and Suesstrunk (2003), as follows:

$$C=\sqrt{\sigma_{\left(R-G\right)}^2+{\sigma}_{\left(\frac{1}{2}\left(R+G\right)-B\right)}^2}+0.3\sqrt{\mu_{\left(R-G\right)}^2+{\mu}_{\left(\frac{1}{2}\left(R+G\right)-B\right)}^2}$$

C is the colorfulness parameter of a pixel in an image, in which R is the red value, G is the green value, and B is the blue value. *σ* represents the standard deviation and *μ* represents the mean. The colorfulness parameters of all pixels were then averaged to get the mean colorfulness of an image. The algorithm showed a correlation of 90% with the data collected from a psychophysical category scaling experiment (Hasler & Suesstrunk, 2003).

In addition, the perceived luminance of each image was computed based on a similar equation proposed by Morgand and Tamaazousti (2014), shown in the following equation.

$$\textrm{L}=\sqrt{0.241{R}^2+0.691{G}^2+0.068{B}^2}$$

L is the luminance parameter of a pixel in an image, in which R is the red value, G is the green value, and B is the blue value. The luminance parameters of all pixels were then averaged to get the mean luminance of an image. After computing the mean colorfulness and luminance of the original images used in all three experiments, the positive images (*M*_C_ = 53.38, *SE*_C_ = 1.30; *M*_L_ = 105.62, *SE*_L_ = 2.53) showed significantly higher mean scores (*p*_C_ < 0.001, *p*_L_ = 0.001) for both colorfulness and luminance than negative ones (*M*_C_ = 46.76, *SE*_C_ = 1.39; *M*_L_ = 94.13, *SE*_L_ = 2.51). Therefore, 19 most colorful positive images and 19 least colorful negative images were excluded to approximately equate the mean colorfulness between the two valence types. Among the remaining 181 images, 13 positive images with the highest luminance and 13 negative images with the lowest luminance were further excluded to approximately equate the luminance levels, leading to 168 images remaining in each valence category. The remaining positive images (*M*_C_ = 50.18, SE_C_ = 1.13; *M*_L_ = 99.98, *SE*_L_ = 2.38) showed approximately the same colorfulness and luminance (*p*_C_ = 0.973, *p*_L_ = 0.921) as negative images (*M*_C_ = 50.12, *SE*_C_ = 1.46; *M*_L_ = 99.63, *SE*_L_ = 2.52). We controlled the colorfulness and luminance of the images in these analyses because we considered them as most relevant to the color mode/saturation comparisons in the experiments.

## Appendix B

**B.1 Experiment 1 results**

Post hoc analyses were then conducted on reduced sets of 168 positive and 168 negative images in Experiment 1, after equating the colorfulness and luminance. A significant interaction was found between valence and color mode (*F*(1, 32) = 60.62, *p* < 0.001). Additionally, results showed significant main effects of valence (*F*(1, 32) = 255.99, *p* < 0.001) and color mode (*F*(1, 32) = 28.56, *p* < 0.001). Paired two-tailed t-tests were conducted for both positive and negative images to confirm the effects. For positive images, chromatic versions (*M* = 34.48, *SEM* = 2.56) had significantly higher ratings than grayscale versions (*M* = 23.67, *SEM* = 2.41) (*t*(32) = 6.83, *p* < 0.001, Cohen’s d_z_ = 1.19). For negative images, chromatic versions (*M* = -43.70, *SEM* = 2.99) had significantly lower ratings than grayscale versions (*M* = -42.06, *SEM* = 3.00) *(t* (32) = -3.30, *p* = 0.002, Cohen’s *d*_z_ = -0.57). These results are consistent with the results reported above before image exclusion.

**B.2 Experiment 2 results**

Post hoc analyses were then conducted on reduced sets of 168 positive and 168 negative images in Experiment 2, after equating the colorfulness and luminance. The results showed a significant interaction between valence and color mode (*F*(1, 32) = 63.88, *p* < 0.001) and significant main effects of valence (*F*(1, 32) = 242.80, *p* < 0.001) and color mode (*F*(1, 32) = 28.39, *p* < 0.001). Positive valence chromatic versions (*M* = 30.12, *SEM* = 1.90) had significantly higher ratings than grayscale versions (*M* = 22.27, *SEM* = 1.92) (*t*(32) = 7.27, *p* < 0.001, Cohen’s d_z_ = 1.27). For negative images, chromatic versions (*M* = -36.02, *SEM* = 3.36) had significantly lower ratings than grayscale versions (*M* = -34.64, *SEM* = 3.27) *(t* (32) = -2.82, *p* = 0.008, Cohen’s *d*_z_ = -0.49). All findings were qualitatively the same as those of data before image exclusion.

**B.3 Experiment 3 results**

Post hoc analyses were then conducted on reduced sets of 168 positive and 168 negative images in Experiment 3, after equating the colorfulness and luminance. The results showed a significant interaction between valence and saturation (*F*(1, 32) = 6.18, *p* < 0.018), and significant main effects of valence (*F*(1, 32) = 199.97, *p* < 0.001) and saturation (*F*(1, 32) = 29.50, *p* < 0.001). For positive images, original chromatic versions (*M* = 31.34, *SEM* = 2.77) had significantly higher ratings than saturation-reduced versions (*M* = 29.41, *SEM* = 2.64) (*t*(32) = 4.43, *p* < 0.001, Cohen’s d_z_ = 0.77). For negative images, original chromatic versions (*M* = -36.20, *SEM* = 2.57) had significantly higher ratings than saturation-reduced versions (*M* = -36.87, *SEM* = 2.63) *(t* (32) = 2.90, *p* = 0.007, Cohen’s *d*_z_ = 0.50). Therefore, here too all findings were consistent with the results from the full set of images before image exclusion.

## References

[CR1] Adams FM, Osgood CE (1973). A cross-cultural study of the affective meanings of color. Journal of Cross-Cultural Psychology.

[CR2] Bekhtereva V, Müller MM (2017). Bringing color to emotion: The influence of color on attentional bias to briefly presented emotional images. Cognitive, Affective, & Behavioral Neuroscience.

[CR3] Boyatzis CJ, Varghese R (1994). Children's emotional associations with colors. The Journal of Genetic Psychology.

[CR4] Bradley MM, Codispoti M, Cuthbert BN, Lang PJ (2001). Emotion and motivation I: Defensive and appetitive reactions in picture processing. Emotion.

[CR5] Bradley MM, Sabatinelli D, Lang PJ, Fitzsimmons JR, King W, Desai P (2003). Activation of the visual cortex in motivated attention. Behavioral Neuroscience.

[CR6] Cano ME, Class QA, Polich J (2009). Affective valence, stimulus attributes, and P300: Color vs. black/white and normal vs. scrambled images. International Journal of Psychophysiology.

[CR7] Castelhano MS, Henderson JM (2008). The influence of color on the perception of scene gist. Journal of Experimental Psychology: Human Perception and Performance.

[CR8] Codispoti M, De Cesarei A, Ferrari V (2012). The influence of color on emotional perception of natural scenes. Psychophysiology.

[CR9] Crozier, W. R. (1999). The meanings of colour: Preferences among hues. *Pigment & resin technology.*

[CR10] Cunningham MK (2017). The value of color research in brand strategy. Open Journal of Social Sciences.

[CR11] Dael N, Perseguers MN, Marchand C, Antonietti JP, Mohr C (2016). Put on that colour, it fits your emotion: Colour appropriateness as a function of expressed emotion. Quarterly Journal of Experimental Psychology.

[CR12] Ettis SA (2017). Examining the relationships between online store atmospheric color, flow experience and consumer behavior. Journal of Retailing and Consumer Services.

[CR13] Gegenfurtner KR, Rieger J (2000). Sensory and cognitive contributions of color to the recognition of natural scenes. Current Biology.

[CR14] Gilbert AN, Fridlund AJ, Lucchina LA (2016). The color of emotion: A metric for implicit color associations. Food Quality and Preference.

[CR15] Goffaux V, Jacques C, Mouraux A, Oliva A, Schyns P, Rossion B (2005). Diagnostic colours contribute to the early stages of scene categorization: Behavioural and neurophysiological evidence. Visual Cognition.

[CR16] Hall RH, Sidio-Hall MA (1994). The effect of color enhancement on knowledge map processing. The Journal of Experimental Education.

[CR17] Hansen T, Gegenfurtner KR (2009). Independence of color and luminance edges in natural scenes. Visual Neuroscience.

[CR18] Hard A, Sivik L (2001). A theory of colours in combination: A descriptive model related to the NCS colour-order system. Color Research and Application.

[CR19] Hasler D, Suesstrunk SE (2003). Measuring colorfulness in natural images. *Human vision and electronic imaging VIII*.

[CR20] Hekkert P (2006). Design aesthetics: Principles of pleasure in design. Psychology Science.

[CR21] Hemphill M (1996). A note on adults' color–emotion associations. The Journal of Genetic Psychology.

[CR22] Humphrey GK, Goodale MA, Jakobson LS, Servos P (1994). The role of surface information in object recognition: Studies of a visual form agnosic and normal subjects. Perception.

[CR23] Humphrey N (2019). The colour currency of nature. *Colour for architecture today*.

[CR24] Hurlbert AC, Ling Y (2007). Biological components of sex differences in color preference. Current Biology.

[CR25] Inoue M, Freel T, Van Avermaete A, Leevy WM (2020). Color enhancement strategies for 3D printing of x-ray computed tomography bone data for advanced anatomy teaching models. Applied Sciences.

[CR26] Jacobs KW, Suess JF (1975). Effects of four psychological primary colors on anxiety state. Perceptual and Motor Skills.

[CR27] Junghöfer M, Bradley MM, Elbert TR, Lang PJ (2001). Fleeting images: A new look at early emotion discrimination. Psychophysiology.

[CR28] Kaufman, A. J., & Lohr, V. I. (2002). Does plant color affect emotional and physiological responses to landscapes?. In *XXVI international horticultural congress: Expanding roles for horticulture in improving human well-being and life quality 639* (pp. 229-233).

[CR29] Kaya N, Epps HH (2004). Relationship between color and emotion: A study of college students. College Student Journal.

[CR30] Kuzinas A, Noiret N, Bianchi R, Laurent É (2016). The effects of image hue and semantic content on viewer’s emotional self-reports, pupil size, eye movements, and skin conductance response. Psychology of Aesthetics, Creativity, and the Arts.

[CR31] Lang PJ, Bradley MM, Cuthbert BN (2008). *International affective picture system (IAPS): Affective ratings of pictures and instruction manual*.

[CR32] Lee CJ, Andrade E (2010). *The effect of emotion on color preferences*.

[CR33] Linhares JMM, Pinto PD, Nascimento SMC (2008). The number of discernible colors in natural scenes. JOSA A.

[CR34] Lu Z, Guo B, Boguslavsky A, Cappiello M, Zhang W, Meng M (2015). Distinct effects of contrast and color on subjective rating of fearfulness. Frontiers in Psychology.

[CR35] Mahnke FH (1996). *Color, environment, and human response: An interdisciplinary understanding of color and its use as a beneficial element in the design of the architectural environment*.

[CR36] Manav B (2017). Color-emotion associations, designing color schemes for urban environment-architectural settings. Color Research & Application.

[CR37] Minns S, Levihn-Coon A, Carl E, Smits JA, Miller W, Howard D, Powers MB (2019). Immersive 3D exposure-based treatment for spider fear: A randomized controlled trial. Journal of Anxiety Disorders.

[CR38] Morgand A, Tamaazousti M (2014). Generic and real-time detection of specular reflections in images. In *2014 international conference on computer vision theory and applications (VISAPP)*.

[CR39] Oliva A, Schyns PG (2000). Diagnostic colors mediate scene recognition. Cognitive Psychology.

[CR40] Ou LC, Luo MR, Woodcock A, Wright A (2004). A study of colour emotion and colour preference. Part I: Colour emotions for single colours. Color Research & Application.

[CR41] Ou LC, Luo MR, Woodcock A, Wright A (2004). A study of colour emotion and colour preference. Part II: Colour emotions for two-colour combinations. Color Research & Application.

[CR42] Ou LC, Luo MR, Woodcock A, Wright A (2004). A study of colour emotion and colour preference. Part III: Colour preference modeling. Color Research & Application.

[CR43] Palmer SE, Schloss KB (2010). An ecological valence theory of human color preference. Proceedings of the National Academy of Sciences.

[CR44] Price CJ, Humphreys GW (1989). The effects of surface detail on object categorization and naming. The Quarterly Journal of Experimental Psychology.

[CR45] Reber R, Schwarz N, Winkielman P (2004). Processing fluency and aesthetic pleasure: Is beauty in the perceiver's processing experience?. Personality and Social Psychology Review.

[CR46] Reber R, Winkielman P, Schwarz N (1998). Effects of perceptual fluency on affective judgments. Psychological Science.

[CR47] Saarela TP, Landy MS (2012). Combination of texture and color cues in visual segmentation. Vision Research.

[CR48] Suk HJ, Irtel H (2010). Emotional response to color across media. *Color Research & Application: Endorsed by inter-society color council, the colour group (Great Britain), Canadian Society for Color, color science Association of Japan, Dutch Society for the Study of color, the Swedish colour Centre Foundation*. Colour Society of Australia, Centre Français de la Couleur.

[CR49] Tanaka J, Weiskopf D, Williams P (2001). The role of color in high-level vision. Trends in Cognitive Sciences.

[CR50] Tanaka JW, Presnell LM (1999). Color diagnosticity in object recognition. Perception & Psychophysics.

[CR51] Valdez P, Mehrabian A (1994). Effects of color on emotions. Journal of Experimental Psychology: General.

[CR52] Wilms L, Oberfeld D (2018). Color and emotion: Effects of hue, saturation, and brightness. Psychological Research.

[CR53] Wurm LH, Legge GE, Isenberg LM, Luebker A (1993). Color improves object recognition in normal and low vision. Journal of Experimental Psychology: Human Perception and Performance.

[CR54] Yendrikhovski SN, Blommaert FJ, de Ridder H (1998). Perceptually optimal color reproduction. *Human vision and electronic imaging III*.

